# Preliminary Effects of Benralizumab in an AML Cell Model with Promyelocytic Features Expressing IL-5R: An Exploratory Proof-of-Concept Study

**DOI:** 10.3390/biomedicines14030652

**Published:** 2026-03-13

**Authors:** Giovanna Lucia Piazzetta, Silvia Di Agostino, Nadia Lobello, Annamaria Aloisio, Anna Di Vito, Jessica Bria, Andrea Filardo, Isabella Coscarella, Mariaimmacolata Preianò, Corrado Pelaia, Nicola Lombardo, Emanuela Chiarella

**Affiliations:** 1 Otolaryngology Head and Neck Surgery, AOU Dulbecco, 88100 Catanzaro, Italy; giovannapiazzetta@hotmail.it (G.L.P.); nadialobello@gmail.com (N.L.); 2 Department of Health Sciences, “Magna Græcia” University of Catanzaro, 88100 Catanzaro, Italy; sdiagostino@unicz.it; 3 Department of Medical and Surgical Sciences, “Magna Græcia” University of Catanzaro, 88100 Catanzaro, Italy; aloisio@unicz.it (A.A.); jessica.bria@studenti.unicz.it (J.B.); filardo@unicz.it (A.F.); preiano@unicz.it (M.P.); pelaia.corrado@unicz.it (C.P.); 4 Department of Experimental and Clinical Medicine, “Magna Græcia” University of Catanzaro, 88100 Catanzaro, Italy; divito@unicz.it (A.D.V.); isabella.coscarella@studenti.unicz.it (I.C.); 5 Department of Pharmacy, Health and Nutrition Sciences, University of Calabria, 87036 Cosenza, Italy; nicola.lombardo@unical.it

**Keywords:** Benralizumab, acute promyelocytic leukemia, IL-5R, STAT, ERK, NF-kB, apoptosis

## Abstract

**Background/Objectives:** Acute myeloid leukemia (AML) comprises a heterogeneous group of diseases, with some subtypes displaying promyelocytic features and altered differentiation programs. Aberrant cytokine receptor signaling has been implicated in leukemogenesis, and IL-5Rα has recently emerged as a potential marker in selected AML subsets, including promyelocytic variants. Benralizumab is a monoclonal antibody directed against the alpha chain of the interleukin-5 receptor (CD125), which blocks IL-5Rα–mediated signaling. This proof-of-concept study aimed to explore the effects of the anti-IL-5Rα monoclonal antibody Benralizumab in an in vitro AML cell model with promyelocytic characteristics. **Methods:** Public transcriptomic datasets were analyzed to evaluate IL-5Rα expression in AML subtypes. HL-60 cells, an AML cell line expressing IL-5Rα, were treated with Benralizumab and analyzed for cell cycle distribution and modulation of key signaling and apoptotic pathways by flow cytometry and Western blotting. **Results:** IL-5Rα was highly expressed in AML, particularly in M2 and M3 subtypes. Benralizumab treatment reduced STAT3 expression, activated ERK and NF-κB signaling, induced p21 and p27 expression, altered cell cycle distribution, and induced caspase-8 cleavage, suggesting activation of extrinsic apoptotic signaling. **Conclusions:** These findings provide preliminary proof-of-concept evidence that IL-5Rα targeting by Benralizumab may directly affect cell survival and cell cycle regulation in AML cells with promyelocytic characteristics. When interpreted together with the in silico analyses performed on AML patient datasets, these results support the rationale for future validation in APL-oriented models carrying the PML::RARα fusion, the disease-defining oncogenic driver generated by the t(15;17) translocation that blocks myeloid differentiation. However, the in silico and in vitro datasets were not formally integrated at the patient level, and these functional results should be considered exploratory.

## 1. Introduction

**Figure 1:**
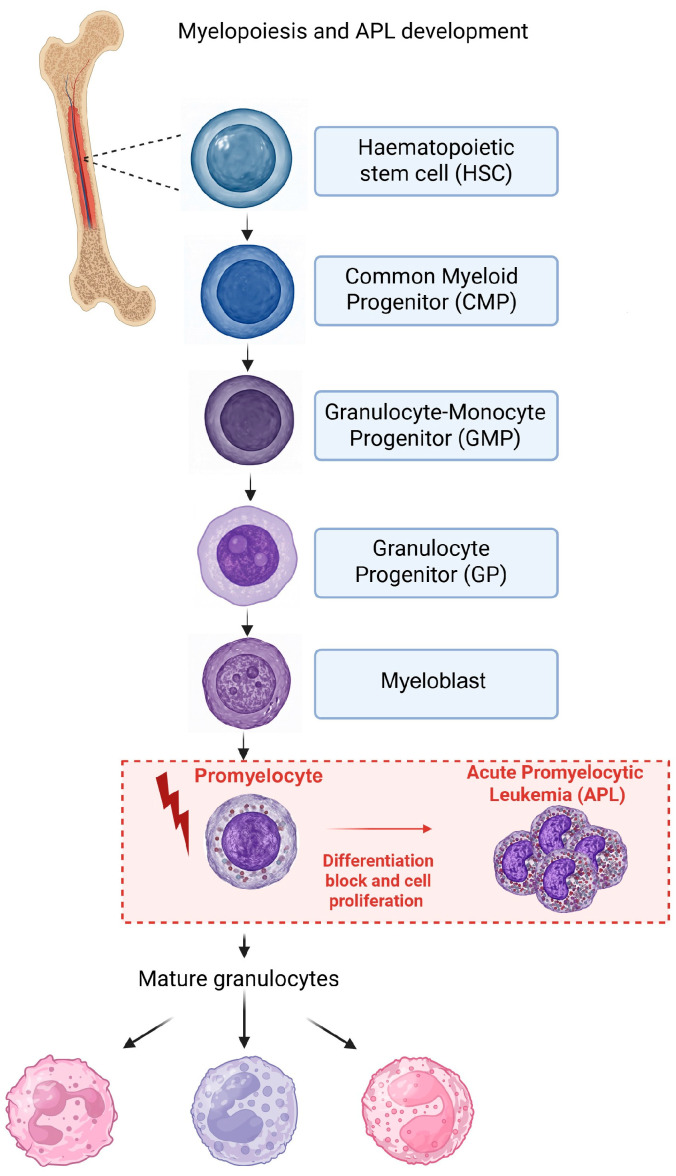
Schematic representation of normal human hematopoiesis and the promyelocytic stage involved in acute promyelocytic leukemia (APL). Hematopoietic stem cells (HSCs) give rise to common myeloid progenitors (CMPs), which further differentiate into granulocyte–monocyte progenitors (GMPs) and subsequently into myeloblasts and promyelocytes along the granulocytic lineage. In APL, the PML::RARα fusion protein induces a differentiation block at the promyelocytic stage, leading to accumulation of immature leukemic cells. Created in BioRender. Chiarella, E. (2026) https://BioRender.com/exkfcn9 (accessed on 3 March 2026).

Acute myeloid leukemia (AML) is a heterogeneous group of hematological malignancies characterized by impaired differentiation and uncontrolled proliferation of myeloid progenitors. AML classification integrates morphology, immunophenotype, cytogenetics, and molecular features [1,2]. An overview of AML classification and diagnostic criteria, including Acute Promyelocitic Leukemia (APL), is provided by established clinical frameworks (https://emedicine.medscape.com/article/2006750-overview) (accessed on 27 February 2026). According to the International Consensus Classification (ICC, 2022) and the WHO 5th edition (2024), AML is broadly divided into categories including: AML with recurrent genetic abnormalities (e.g., promyelocytic leukemia protein–retinoic acid receptor alpha (PML::RARα) in APL), AML with myelodysplasia-related changes, therapy-related AML, and AML not otherwise specified (NOS) based on morphologic and molecular features [3,4,5]. AML can be further subclassified by differentiation stages—promyelocytic, myeloblastic, and monocytic—reflecting the maturation arrest of the leukemic clone. APL represents a distinct genetic and morphologic entity characterized by PML::RARα fusion and promyelocytic differentiation arrest, which historically provided the basis for differentiation therapy using ATRA and arsenic trioxide (ATO) [6]. The hierarchical organization of normal human hematopoiesis and the promyelocytic stage at which APL arises are illustrated in Figure 1.

ATRA, a vitamin A derivative, is an effective differentiation-inducing agent in AML and other malignancies [7]. Treatment with ATRA yields complete remission in 80–90% of patients with newly diagnosed APL by stimulating the differentiation of the immature leukemia cells [8,9]. However, ATRA monotherapy often leads to relapse due to acquired resistance, highlighting the need for combination with chemotherapy or arsenic trioxide (ATO) [10,11]. Therefore, ATRA treatment is usually combined with anthracycline-based chemotherapy or ATO [10,12,13].

These limitations have prompted investigation of complementary targets, including the cytokine receptor IL-5Rα, which may modulate leukemic cell behavior. Although interleukin-5 receptor alpha (IL-5Rα) has been primarily studied in the context of eosinophils, recent transcriptomic analyses have identified IL5RA expression in specific AML subsets, notably in t(8;21) AML, where single-cell profiling revealed enrichment of IL5RA transcripts in leukemic stem and blast populations, supporting a potential involvement of IL-5-related signaling in leukemic biology [14]. This suggests deregulated IL-5Rα activity beyond eosinophils [14]. Moreover, IL5RA has emerged in recent transcriptomic analyses as part of immune-related gene signatures linked to outcome variability in AML, although its independent prognostic impact remains to be clarified [15,16].

HL-60 cells, originally derived from a patient with APL but lacking the PML::RARα fusion, are classified as FAB-M2 and serve as a model of promyelocytic-like AML. Unlike normal hematopoiesis, HL-60 cells retain developmental plasticity and can differentiate into granulocytes, eosinophils, monocytes, or macrophages, depending on the stimulus (e.g., DMSO, PMA, TPA, butyrate, actinomycin D, or retinoic acid) [17,18,19,20,21,22]. IL-5Rα expression in HL-60 cells correlates with eosinophilic commitment and is dynamically regulated by external stimuli, making this cell line a relevant in vitro model to study IL-5Rα-dependent signaling and its modulation by targeted therapies in immature myeloid leukemias [17,18,19,20,21,22].

IL-5 exerts its effects by binding to the heterodimeric IL-5 receptor (IL-5R), composed of the α chain (IL-5Rα) and the shared βc subunit. Ligand binding induces receptor dimerization and activation of JAK1 and JAK2, leading to phosphorylation of tyrosine residues that recruit downstream signaling molecules [23].

The main pathway is JAK/STAT, particularly STAT5, which dimerizes and translocates to the nucleus to regulate genes controlling eosinophil differentiation, survival, activation, and cytokine production. IL-5 also activates the PI3K/AKT pathway, promoting survival and metabolism, and the RAS/RAF/MEK/ERK (MAPK) pathway, supporting survival, proliferation, and functional activation, and engages NF-κB to promote transcription of pro-survival and inflammatory genes [23,24,25]

**Figure 2:**
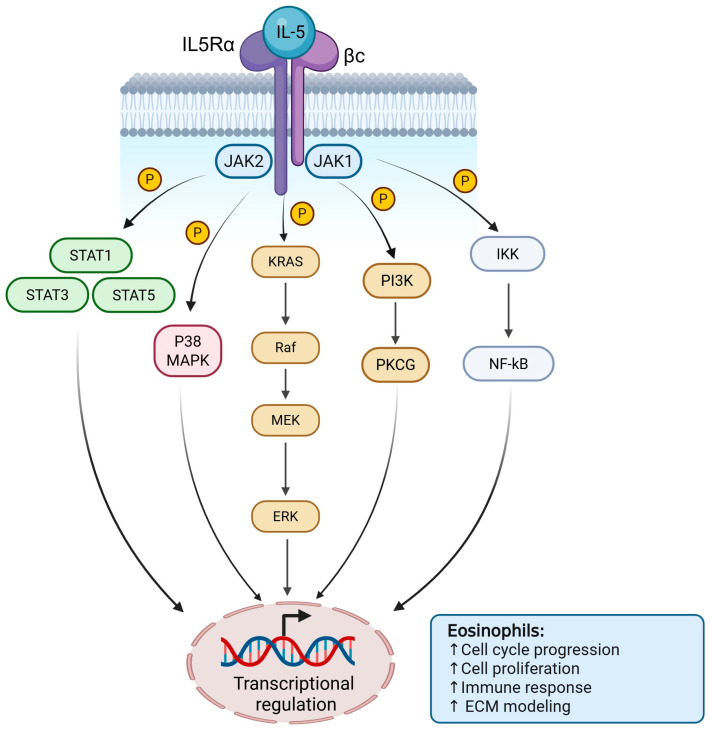
Schematic representation of IL-5/IL-5R signaling pathway. Binding of IL-5 to its heterodimeric receptor (IL-5Rα and βc subunits) induces activation of receptor-associated JAK kinases, leading to phosphorylation of STAT5 and its nuclear translocation. Parallel activation of the PI3K/AKT and MAPK (RAS/RAF/MEK/ERK) pathways contributes to eosinophil survival, proliferation, activation, and resistance to apoptosis. The integrated signaling network sustains eosinophilic inflammation and type 2 immune responses. Created in BioRender. Chiarella, E. (2026) https://BioRender.com/89trve5 (accessed on 3 March 2026).

Additionally, IL-5 signaling enhances cytoskeletal changes, adhesion, and apoptosis resistance, sustaining eosinophil tissue infiltration and type 2 inflammation [26]. A schematic of these molecular interactions is shown in Figure 2.

In acute leukemias, dysregulated JAK–STAT signaling contributes substantially to leukemogenesis and disease progression, emerging as a relevant therapeutic target [27]. The specification of myeloid progenitor cell fate depends on various STAT3 and STAT5 isoforms and the cellular context [28,29]. Dysregulation of JAK-STAT signaling is implicated in the pathogenesis and tumor progression of multiple cancers including myeloid malignancies. STAT3 is constitutively active in ~50% of AML cases, while STAT5 is modulated in FLT3-ITD AML [14]. Aberrant STAT3 and STAT5 signaling promotes proliferation and apoptosis resistance, while STAT3 inhibition induces apoptosis and reduces proliferation in HL-60 cells [28,30,31,32,33,34]. In addition to JAK–STAT, the MAPK/ERK pathway regulates proliferation, differentiation, and stress responses in myeloid leukemia cells [35,36,37].

The mitogen-activated protein kinase (MAPK) pathway activation is a recognized feature of ATRA-induced HL-60 differentiation, contributing to growth arrest and maturation [38]. Activation of the ERK pathway was observed in the differentiation stages of HL-60 cells exposed to Xestospongin C (XC) (500 nM), and this effect was reversed by administration of the ERK inhibitor PD98059 [39]. Intriguingly, the ERK pathway activation was also related to ER stress in HL-60 cells induced to differentiate into neutrophil-like cells (dHL60) by stimulation with PMA, a pharmacologic NADPH oxidase activator [40]. These findings support the relevance of ERK signaling in controlling leukemic cell fate decisions. In parallel, NF-κB signaling, a critical mediator of myeloid differentiation, apoptosis, and stress responses, is frequently aberrantly activated in AML, including in primary leukemic cells, where its activation correlates with reduced apoptosis, enhanced survival, and chemoresistance, thereby contributing to leukemogenesis and therapeutic resistance [41,42]. Finally, deregulation of apoptotic pathways is a hallmark of leukemia cell survival and drug resistance. In HL-60 cells, caspase-8 acts as an upstream activator of apoptosis, and several compounds trigger cell death via this pathway, highlighting extrinsic apoptosis as a potential therapeutic target [43,44,45].

In this study, we investigated the effects of the IL-5Rα–targeting monoclonal antibody Benralizumab in HL-60 cells, an AML model with promyelocytic-like features, focusing on its modulation of signaling pathways, cell cycle, and apoptosis. We combined in silico transcriptomic analyses with in vitro functional validation to assess IL-5Rα inhibition as a complementary strategy to modulate survival and differentiation in immature myeloid leukemias, providing a rationale for potential therapeutic targeting of IL-5Rα in promyelocytic AML.

## 2. Materials and Methods

### 2.1. In Silico Analysis

The expression of IL5RA in AML patients was analyzed using the TCGA database via the GEPIA2 tool (http://gepia2.cancer-pku.cn/#index) (accessed on 10 January 2026), applying a *p*-value threshold of 0.05. Pair-wise correlation analyses (Spearman and Pearson) were performed on RNA-seq data from the AML Oregon Health & Science University (OHSU) dataset (942 patients) retrieved from cBioPortal (https://www.cbioportal.org) (accessed on 20 November 2025), using log-scale visualization and *p* < 0.05 as the significance threshold. ROC curve analyses integrating transcriptomic and drug sensitivity data from leukemia cell lines were performed using ROCplot (https://rocplot.com/cells) (accessed on 10 January 2026), and the area under the curve (AUC) with 95% confidence intervals was calculated. ROCplot analyses were used as an exploratory, hypothesis-generating approach based on publicly available datasets. Therefore, results should be interpreted with caution due to dataset heterogeneity, variable experimental conditions across cell lines, and potential batch effects.

The BloodSpot tool (https://www.fobinf.com) (accessed on 10 January 2026), was used to assess IL-5RA expression across normal hematopoietic lineages and AML patient samples. Specifically, the “Leukemia Dataset” was selected to visualize IL-5RA expression in different AML subtypes and stages of myeloid differentiation. Gene expression levels were normalized and displayed as log2-transformed values. Boxplots and expression profiles were generated within the BloodSpot interface, and the resulting data were exported for further analysis (Supplementary Table S1). This approach allowed us to compare IL-5RA expression between immature myeloid progenitors and differentiated lineages, providing context for the relevance of IL-5RA in AML.

Additionally, gene correlation analyses in AML patients were conducted using cBioPortal to identify co-expressed genes potentially involved in IL-5Rα-dependent signaling pathways. These in silico analyses were designed to explore the relevance of IL-5Rα expression in AML and its potential association with pathways implicated in leukemic cell survival, proliferation, and differentiation.

### 2.2. Cell Culture and Pharmacological Treatment

HL-60 obtained from the American Type Culture Collection (ATCC-CCL-240) were cultured at 1.0–2.0 × 10^5^ cells/mL in RPMI 1640 medium (Life Technologies, Carlsbad, CA, USA, 11875-093) containing 10% fetal bovine serum (FBS) (Life Technologies, Carlsbad, CA, USA, 10270-106) and 1% penicillin/streptomycin solution (Life Technologies, Carlsbad, CA, USA). Cells were maintained at 37 °C in a humidified atmosphere of 5% CO_2_. Cells were exposed to Benralizumab at the concentrations of 5 and 50 µM daily for 3 d and then analyzed [46]. The concentrations selected for this in vitro study were based on preliminary dose–response screening aimed at identifying conditions capable of inducing measurable modulation of intracellular signaling pathways in HL-60 cells. Given the static culture system and absence of immune effector components, these concentrations should be interpreted as experimental conditions to explore direct receptor-associated signaling effects rather than as a simulation of clinical pharmacokinetics. Benralizumab was kindly provided by AstraZeneca (Cambridge, UK) under a material transfer agreement for research purposes only, with no role of the company in study design, data analysis, or interpretation.

The concentrations used in this exploratory in vitro study do not reflect clinically achievable plasma levels of Benralizumab. High micromolar concentrations were selected to ensure sufficient receptor occupancy under static culture conditions lacking immune effector components (e.g., ADCC). Therefore, these doses should be interpreted as mechanistic saturation conditions aimed at evaluating direct signaling modulation rather than pharmacokinetic simulation of clinical exposure.

### 2.3. Cell Cycle Analysis

For the cell cycle analysis [47], 2.0 × 10^5^ cells were fixed dropwise in ice-cold 70% ethanol (Sigma-Aldrich, St. Louis, MO, USA) for 30 min in the dark at 4 °C. After three washes in cold PBS (Life Technologies, Carlsbad, CA, USA), HL-60 cells were treated with RNase (Sigma-Aldrich) (40 μg/mL) for 30 min at room temperature and then stained with propidium iodide (PI) (Sigma-Aldrich, St. Louis, MO, USA) (50 μg/mL) for 10 min at room temperature. Cells were acquired on the BD FACScan™ II (BD Biosciences, San Jose, CA, USA) for analysis using a 488 nm excitation. Flow cytometry data were analyzed using FlowJo software version 8.8.6 (BD Biosciences, Ashland, OR, USA). 

### 2.4. Total Protein Extraction

HL-60 cells cultured in the presence of Benralizumab (5 and 50 μM) for 72 h were collected by centrifugation at 1000 revolutions per minute (rpm) for 5 min and then resuspended in lysis buffer (250 mM Tris-HCl pH 7.5) (Sigma-Aldrich) added with protease inhibitors (Sigma-Aldrich, St. Louis, MO, USA, P8340). After three freeze–thaw cycles (−80–37 °C), the samples were centrifuged at 12,000 rpm for 10 min at 4 °C. The supernatants were recovered, and protein concentrations were determined by the Bradford assay (Bio-Rad Laboratories, Hercules, CA, USA) [48].

### 2.5. Western Blot

Western blot analysis was performed as previously described [49]. Briefly, 25 µg of total protein extracts was electrophoretically separated under reducing conditions on 4–12% NuPAGE Novex bis-Tris gradient polyacrylamide gels (Life Technologies, Carlsbad, CA, USA, NP0322BOX) and then electroblotted onto nitrocellulose filters. The membranes were blocked with 5% non-fat milk (Bio-Rad Laboratories, Hercules, CA, USA) and incubated with primary antibodies. Anti-STAT5a (Santa Cruz Biotechnology, Dallas, TX, USA, sc-1081), anti-STAT-3 (Santa Cruz Biotechnology, Dallas, TX, USA, sc-8019), anti-p-27 (Santa Cruz Biotechnology, Dallas, TX, USA, sc-56454), anti-p-21 (Sigma-Aldrich, St. Louis, MO, USA, P1484), anti-Bcl2 (Santa Cruz Biotechnology, Dallas, TX, USA, sc-492), anti-p-ERK (Cell Signaling Technology, Danvers, MA, USA, 9105), anti-ERK1/2 (Cell Signaling Technology, Danvers, MA, USA, 91025) and antibodies were used at a 1:5000 dilution; anti-NFκB p65 (Santa Cruz Biotechnology, Dallas, TX, USA, sc-372) was used at a 1:1000 dilution; and anti-actin antibody (Santa Cruz Biotechnology, Dallas, TX, USA, sc-8432) was used at a 1:12,000 dilution. Antibody binding was detected using the horseradish peroxidase (HRP)-conjugated goat anti-rabbit IgG or anti-mouse IgG (Agilent Technologies, Santa Clara, CA, USA, P0448). The HRP signal was revealed as chemiluminescence using the ImmunoCruz Western blotting luminal reagent (Santa Cruz Biotechnology, Dallas, TX, USA). Western blot densitometry was determined using the NIH Image J software 1.49v. Density histograms of protein expression were normalized to β-actin levels [49]. Western blot analyses were performed at concentrations selected based on preliminary dose–response screening, focusing on conditions showing clear pathway modulation. Densitometric analysis of Western blot bands was performed using QuantityOne 4.6.8 software (Bio-Rad Laboratories, Hercules, CA, USA).

### 2.6. Statistical Analysis

Data are presented as mean ± standard deviation (SD). Statistical analyses were performed using GraphPad Prism 8 (GraphPad Software, San Diego, CA, USA). For in vitro experiments, differences between treated and control groups were evaluated using unpaired two-tailed Student’s *t*-test. For bioinformatic analyses, non-parametric tests were applied as appropriate: the Mann–Whitney U test was used for ROCplot-based comparisons, and the Wilcoxon rank-sum test was used for TCGA dataset analyses. A *p*-value < 0.05 was considered statistically significant (* *p* < 0.05; ** *p* < 0.01; *** *p* < 0.001; **** *p* < 0.0001). All Western blot experiments were performed in at least three independent biological replicates.

## 3. Results

### 3.1. Expression of IL-5RA in AML

**Figure 3:**
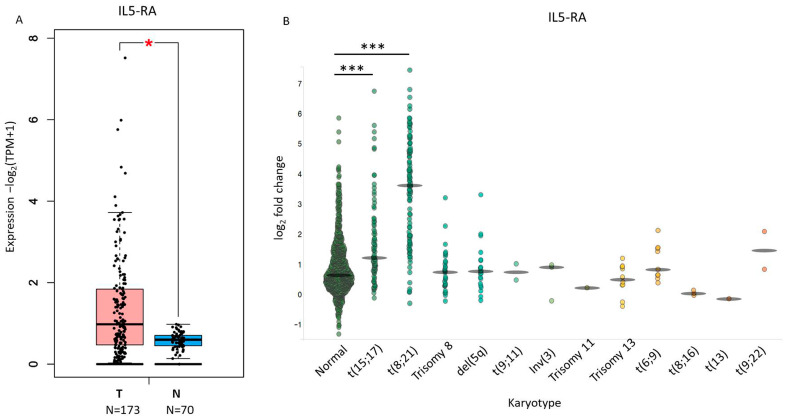
Expression and clinical relevance of IL-5RA in AML. (**A**) Boxplot showing gene expression changes in transcript per million (TPM) of IL-5RA in AML samples (T) versus normal blood samples (N) from the TCGA-AML dataset. The *p* value calculated with a Wilcoxon rank-sum test are shown; * *p* < 0.05. (**B**) Scatter plot showing the expression pattern of IL-5RA in diverse AML subtypes showing chromosomal aberrations. Each data point represents an individual patient (scatter plot format), and the central flattened ellipse indicates the median value for each subtype. *** *p* < 0.001. Abbreviations are defined in Supplementary Table S1.

In order to evaluate the expression of IL-5RA in samples from AML patients with particular attention to AML subtypes characterized by immature promyelocytic features, we considered the public TCGA dataset of AML. We observed that it was significantly expressed at high levels in AML patients compared to control blood cells (Figure 3A). Next, using AML datasets from BloodSpot software, we determined the expression pattern of IL-5RA in AML with genetic abnormalities (gene or chromosome alterations) compared to AML samples with a normal karyotypic structure (Figure 3B, Supplementary Table S1). The subgroup with t(8;21) alteration (AML1-ETO) is more commonly associated with the M2 subtype of AML, while the APL subgroup defined by the characteristic translocation t(15;17) (PML-RARA) is predominantly found in the M3 subtype [50]. Interestingly, the highest levels of IL5RA are significantly associated with t(8;21) and t(15;17) subgroups, as reported in Figure 3B (see legend in the Supplementary Table S1). These alterations are respectively associated with the FAB-M2 and FAB-M3 AML subtypes, suggesting that IL-5Rα overexpression is enriched in AML entities sharing promyelocytic differentiation arrest rather than being restricted to a single cytogenetic subtype. We further analyzed another AML dataset (OHSU) [51] from the cBioPortal where we conducted the analysis on 451 samples with the available data (Supplementary Figure S1). In a similar way, we reported the association of higher levels of IL5RA in the AML1-ETO t(8;21) subgroups (M2) and in APL/PML-RAR*α* t(15;17) (M3) compared to other subtypes.

**Figure 4:**
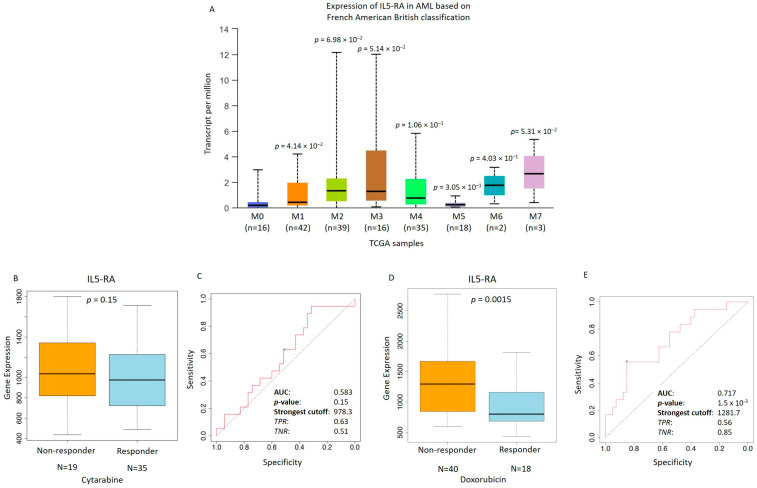
IL-5RA expression across FAB AML subtypes and its association with chemotherapy response. (**A**) IL-5RA mRNA expression analyzed with respect to FAB subgroup classification of AML (TCGA dataset). Significant *p* values: MO vs. M1, *p* = 4.14 × 10^−2^; MO vs. M2, *p* = 6.98 × 10^−2^; MO vs. M3, *p* = 5.14 × 10^−2^; MO vs. M4, *p* = 1.06 × 10^−1^; MO vs. M5, *p* = 3.05 × 10^−1^; MO vs. M6, *p* = 4.03 × 10^−1^, and MO vs. M7, *p* = 5.31 × 10^−2^. (**B**) Box plot showing IL5-RA gene expression levels in Cytarabine non-responder (N = 19) and responder (N = 35) groups. Statistical significance was assessed by Mann–Whitney test. (**C**) ROC curve evaluating the predictive performance of IL5-RA expression for Cytarabine response showing limited accuracy (AUC = 0.583; *p* = 0.15). For panels (**A**,**B**,**D**), box plots represent the median (central line), the interquartile range (box), and the minimum and maximum values (whiskers). Error bars indicate standard deviation (SD) where applicable. (**D**,**E**) Box plot comparing IL5-RA gene expression levels between Doxorubicin non-responder (N = 40) and responder (N = 18) groups. Statistical significance was assessed by Mann–Whitney test. ROC curve demonstrating the predictive value of IL5-RA expression for Doxorubicin treatment response in a panel of leukemia cell lines (AUC = 0.717). The optimal cutoff value (1281.7) is indicated together with the true positive rate (TPR = 0.56) and true negative rate (TNR = 0.85). Statistical significance was assessed by Mann–Whitney test (*p* = 0.0081). Analysis was performed with https://rocplot.com/cells (accessed on 10 January 2026).

Accordingly, examination of the FAB subgroups from the TCGA dataset highlighted that the M2 and M3 subtypes showed the highest levels of IL5-R mRNA expression, considering the low number of patients in the other subgroups. This suggests a correlation between elevated IL-5Rα expression and AML subtypes with immature promyelocytic features including both FAB-M2 and FAB-M3 entities (Figure 4A). Although AML1-ETO is typically associated with the M2 subtype, the high IL-5Rα expression observed both in AML1-ETO-positive cases and in the M3 FAB subgroup suggests that IL-5Rα upregulation may reflect an immature promyelocytic phenotype rather than a single cytogenetic lesion, marking a common biological feature across distinct AML subtypes.

Finally, the predictive value of IL5-RA expression was assessed entirely in silico for the two chemotherapy agents, Cytarabine and Doxorubicin, in a panel of leukemia cells to broaden the clinical relevance of the analysis. Cytarabine is a key component of the standard “7+3” induction regimen for AML [52]. Cytarabine-treated cells that were resistant to treatment (non-responders) tended to have higher IL5-RA levels than sensitive cells (responders), likely reflecting the limited number of available cells (Figure 4B). Consequently, ROC analysis revealed limited predictive accuracy (AUC = 0.583, *p* = 0.15), indicating a weak and exploratory association. In contrast, Doxorubicin sensitivity showed a statistically significant but moderate predictive value (Figure 4C). Furthermore, IL5-RA expression demonstrated a stronger predictive value for response to Doxorubicin. Sensitive cell lines showed significantly lower IL5-RA levels than non-responders with a robust ROC curve (AUC = 0.717, *p* = 1.5 × 10^−5^) (Figure 4D,E). Consistent with these findings, cells expressing high levels of IL5-RA showed reduced sensitivity to chemotherapy, suggesting that IL5-RA could represent a candidate for further study with Benralizumab. These observations suggest a potential role for IL-5Rα targeting in AML subsets with high receptor expression and warrant further investigation. Taken together, these results indicate that IL-5Rα expression is enriched in AML subtypes with promyelocytic features and may represent both a prognostic marker and a potential therapeutic target, providing the rationale for subsequent functional and translational studies.

### 3.2. Cell Cycle Distribution of HL-60 Cells Exposed to Benralizumab Treatment

To examine the impact of Benralizumab on HL-60 cells, a cell cycle analysis was conducted. HL-60 cells were treated with concentrations of 1 nM, 10 nM, 100 nM, 1 μM, and 5 μM Benralizumab for 3 d. Subsequently, PI staining was utilized to assess the distribution of cells across different phases of the cell cycle.

**Figure 5:**
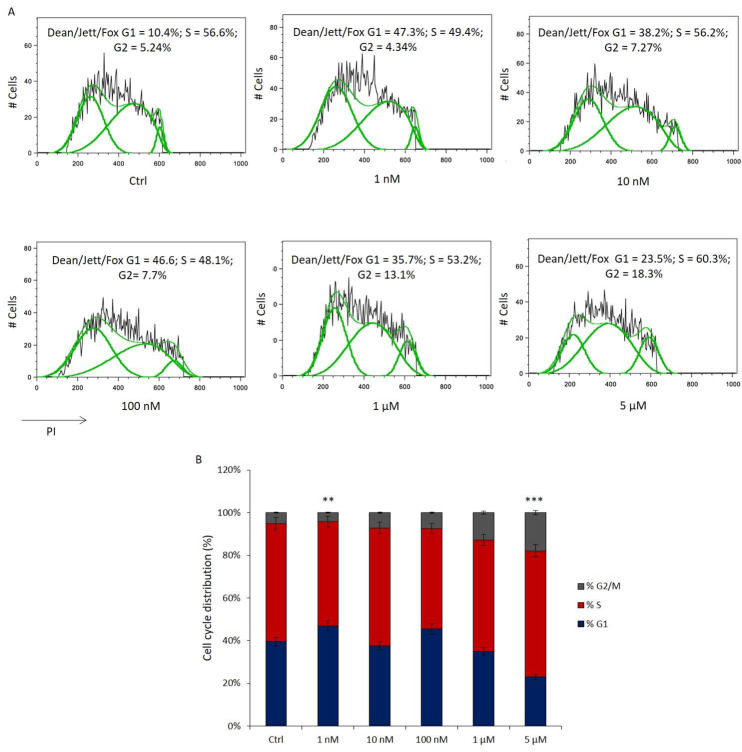
The effect of Benralizumab on cell cycle of HL-60 cells. (**A**,**B**) Cell cycle distribution was analyzed in HL-60 cells exposed to 1 nM, 10 nM, 100 nM, 1 μM, and 5 μM Benralizumab for 72 h, compared with untreated control cells. Dean–Jett–Fox analysis was used to evaluate DNA content and assign cells to G0/G1, S, and G2/M phases. DNA was stained with propidium iodide (PI). Quantitative assessment of the percentages of cells in G0/G1, S, and G2/M phases is shown. No significant variations were observed at lower concentrations (1–100 nM), while higher concentrations (1 and 5 μM) induced a reduction in G0/G1 cells and a concomitant increase in the G2/M phase. Data represent mean ± SD of three independent experiments. ** *p* < 0.01, *** *p* < 0.001 *n* = 3 independent biological experiments per condition.

At lower concentrations (1–100 nM), Benralizumab did not induce significant changes in the cell cycle distribution of HL-60 cells (Figure 5A). In contrast, treatment with higher concentrations (1–5 µM) resulted in a dose-dependent reduction in cells in the G0/G1 phase, accompanied by a progressive increase in the G2/M population (Figure 3B). However, these changes did not reach statistical significance and should be interpreted as trends rather than definitive effects. In particular, a tendency toward increased S-phase and G2/M accumulation was observed at 5 µM Benralizumab, suggesting a possible perturbation of DNA synthesis and cell cycle progression. These preliminary results suggest that Benralizumab may modulate HL-60 proliferation at higher doses, providing a rationale for future functional studies to distinguish cytostatic from cytotoxic effects.

Under these experimental conditions, cell cycle distribution was assessed using the Dean–Jett–Fox analysis model for DNA histogram deconvolution, a widely accepted algorithm for flow cytometric cell cycle analysis [53].

### 3.3. Benralizumab Modulates IL-5R Signals Transduced Through STATs, NF-kB, and ERK Signaling Pathways

**Figure 6:**
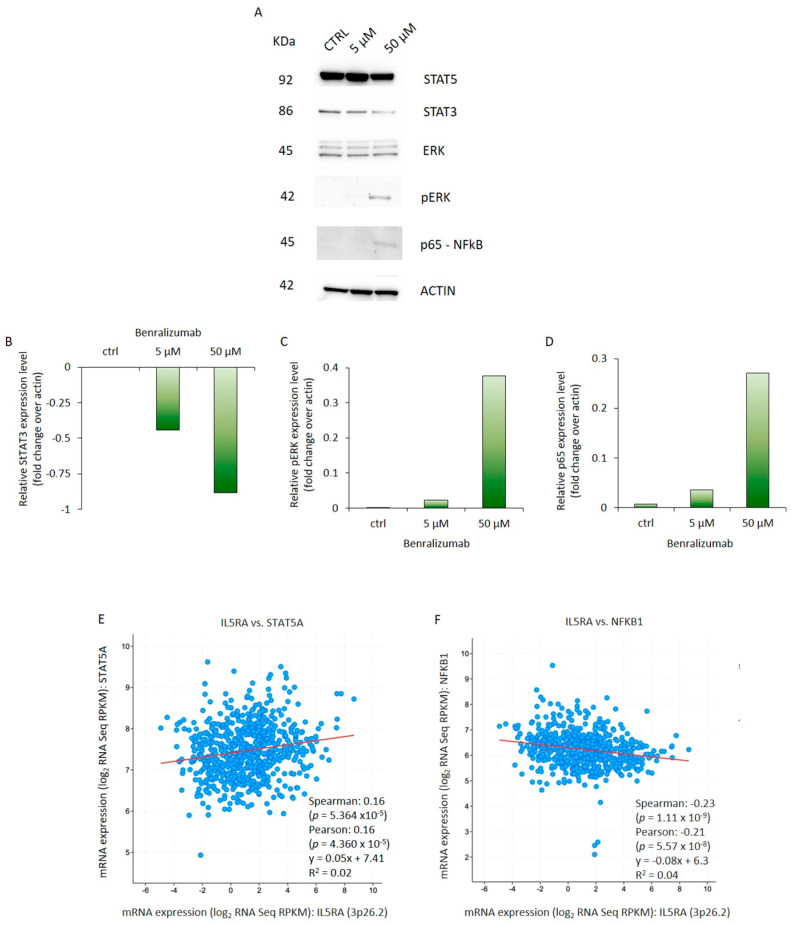
Benralizumab alters the signals transduced through the STATs, NF-kB, and ERK signaling pathways mediated by IL-5R. (**A**) Total extracts from HL-60 cells exposed to different concentrations of Benralizumab (1 nM, 10 nM, 100 nM, 1 μM, and 50 μM) were analyzed by Western blotting for STAT5, STAT3, ERK, pERK, and p65 expression respectively. Actin was used as a control for the amounts of extract loaded. (**B**–**D**) Relative expression level for each protein was measured by QuantityOne software. *n* = 3 independent biological experiments per condition. (**E**,**F**) Pearson and Spearman correlation coefficient and *p* value between IL-5 receptor mRNA expression and STAT5 (**E**) and NF-kB (**F**) in a cohort of 805 AML patients (942 specimens from cBioPortal) analyzed by RNA-seq [54]. Gene expression levels are reported as log2 of reads per kilobase of transcript per million mapped reads (RPKM). Linear regression analysis is shown, wherein R^2^ represents the coefficient of determination, indicating the goodness of fit of the regression model.

It has been established that IL-5R expression is a feature of a subset of AML subtypes. To constitutively block activated IL-5R, Benralizumab was added to the culture medium of the human promyeloblast cell line HL-60. Specifically, HL-60 cells were treated daily for 3 d with the monoclonal antibody targeting the IL-5 receptor at concentrations of 5 and 50 μM to assess the dose-dependent molecular effects of the drug. The three major signaling pathways activated by IL-5R were investigated. To this end, we observed that Benralizumab treatment failed to modulate STAT5 expression in HL-60 cells (Figure 6A,B). Conversely, STAT3 protein levels significantly decreased when cells were treated three times with Benralizumab, especially at the concentration of 50 µM (Figure 6A,B). Interestingly, Benralizumab incubation was able to induce p-ERK and p65 protein expression in HL-60 cells. p-ERK and p65 protein levels were detected only in the cells treated with Benralizumab at the maximum concentration compared to both the control and cells treated with Benralizumab at 5 µM (Figure 6A,C,D). In addition, exploratory correlation analyses in AML patient datasets between IL-5RA and STAT5 or NF-kB expression suggested a trend consistent with the mechanistic observations in HL-60 cells (Figure 6E,F). However, the observed Spearman correlation coefficients were weak, despite reaching statistical significance, indicating limited biological strength of association. These analyses were purely in silico and intended to provide contextual support rather than mechanistic proof. In line with the in vitro findings, STAT5 protein levels were not significantly modulated by Benralizumab treatment.

Nevertheless, validation in larger patient cohorts will be necessary to confirm these potential relationships.

### 3.4. Administration of Benralizumab Results in Heightened Expression of Genes Associated with Cell Cycle and Apoptosis in HL-60 Cells

**Figure 7:**
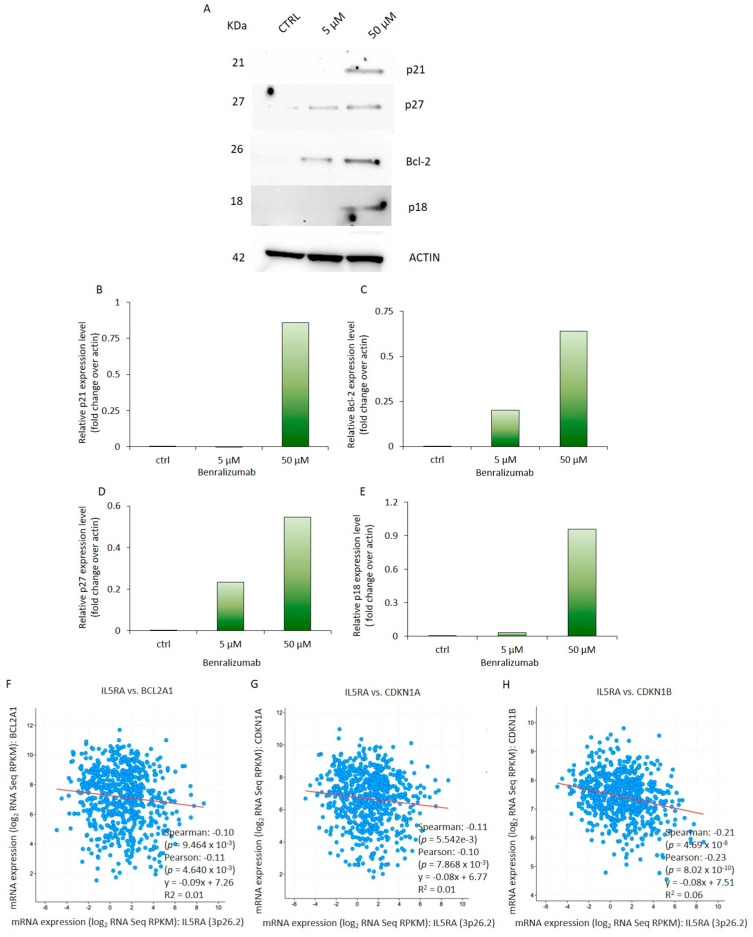
Treatment with Benralizumab results in the upregulation of cell cycle and apoptosis genes in HL-60 cells. (**A**) Whole-cell extracts were subjected to Western blot analysis to assess the expression of p21, p27, Bcl2, and p18, respectively. Actin is shown as a loading control. Please note that the actin blot is the same as that presented in Figure 3 as these proteins were analyzed within the same experimental run and Western blotting session. (**B**–**E**) The QuantityOne software was utilized to measure the relative expression level for each protein. *n* = 3 independent biological experiments per condition. (**F**–**H**) Pearson and Spearman correlation coefficient and *p* value between IL-5 receptor mRNA expression and that of Bcl2 (**F**), p21 (CDKN1A) (**G**), and p27 (CDKN1B) (**H**) in a cohort of 805 AML patients (942 specimens available data from cBioPortal) analyzed by RNA-seq (available data from cBioPortal) [54]. RPKM, reads per kilobase million. Protein expression data (**A**–**E**) are derived from in vitro HL-60 experiments, whereas correlation analyses (**F**–**H**) are based on independent in silico AML patient datasets. These datasets are not directly linked and should be interpreted separately.

To further delineate the molecular effects of Benralizumab on the HL-60 cell cycle, we assessed the expression of key regulators involved in cell cycle progression and apoptosis. Intriguingly, expression of the p21 protein was exclusively observed in HL-60 cells exposed to 50 µM Benralizumab (Figure 7A,B), with no signal detected in control cells or those exposed to 5 µM of Benralizumab. Moreover, p27 protein levels exhibited approximately a two-fold significant increase in HL-60 cells treated with 5 μM Benralizumab and four-fold in cells treated with 50 μM Benralizumab compared to control cells (Figure 7A–D). These findings align with the role of p21 and p27 factors which, upon induction by anti-mitogenic signals and DNA damage, facilitate cell cycle arrest by inhibiting cyclin-cyclin-dependent kinase (CDK) complexes [55]. Additionally, we demonstrated a dose-dependent increase in Bcl-2 expression upon Benralizumab treatment. Indeed, Bcl-2 protein levels were elevated by two-fold and six-fold in cells exposed to 5 and 50 µM of the monoclonal antibody, respectively, compared to untreated cells (Figure 7A–E). Lastly, expression of the Caspase-8/CASP8 p18 subunit (cleaved active form), recognized as a pivotal regulator of cell death pathways [56], was solely detected in cells treated for 3 d with 50 µM of Benralizumab, suggesting the involvement of Caspase-8/CASP8 p18 subunit (cleaved active form) in promoting apoptosis under high-concentration Benralizumab treatment (Figure 7A–E). Nevertheless, these findings should be interpreted with caution. While Caspase-8/CASP8 p18 subunit (cleaved active form) induction suggests activation of the extrinsic apoptotic pathway, the Bcl-2 upregulation may represent a compensatory stress response, highlighting the complexity of apoptotic regulation in vitro [57].

Overall, these findings underscore the potential role of Caspase-8/CASP8 p18 subunit (cleaved active form) in mediating the activation of extrinsic apoptotic signaling upon Benralizumab exposure. Comprehensive analysis of both intrinsic and extrinsic apoptosis markers will be necessary in future studies to fully elucidate the apoptotic mechanisms induced by Benralizumab.

Furthermore, expression of IL-5 receptor exhibited an inverse correlation with the mRNA levels of Bcl-2, p21, and p27 in AML patients, as determined by Spearman and Pearson correlation analyses (Figure 7F–H). The protein expression data obtained in HL-60 cells represent in vitro experimental findings, whereas the correlation analyses shown in Figure 7 are derived from independent in silico AML patient datasets. These two datasets are not directly linked and should therefore be interpreted separately. Moreover, the observed Spearman correlation coefficients were weak, despite statistical significance, indicating limited strength of association.

## 4. Discussion

Resistance to differentiation therapy and uncontrolled proliferation of immature myeloid cells remain critical hurdles in AML treatment. In this study, we show that treatment of HL-60 cells with Benralizumab modulates STAT3, ERK, and NF-κB signaling and alters cell cycle checkpoint proteins, with changes that may contribute to activation of extrinsic apoptotic pathways. These findings provide preliminary evidence that IL-5Rα inhibition may influence pathways relevant to differentiation-based therapies and warrants further investigation.

Here, we investigated the anti-leukemic effect exerted by Benralizumab, an IL-5R inhibitor commonly used in the treatment of eosinophilic asthma [58]. To address this rationale, we focused on molecular aspects in the lineage-uncommitted human myeloblastic cell line HL-60 exposed to Benralizumab treatment for 3 d. These cells closely resemble early myeloid progenitors. They have been considered since the 1970s as a valuable experimental model for studying the molecular basis of leukemogenesis in AML with promyelocytic features F—H and for investigating new drugs capable of modulating differentiation-related signaling pathways [38,59]. Specifically, the levels of STAT3/STAT5, pERK as a readout of MAPK pathway activation, and NF-κB (p65) [24] were studied in HL-60 cells treated with 5 and 50 μM of Benralizumab for 3 d. Total ERK protein levels remained unchanged, but pERK markedly increased at the highest concentration, indicating MAPK/ERK pathway activation rather than a change in total ERK.

IL5RA was primarily investigated at the transcriptomic level in silico to establish its relevance across AML subtypes with promyelocytic features. In vitro analyses were therefore focused on downstream signaling effectors of IL-5Rα activation (STAT3, STAT5, ERK, and NF-κB) as functional readouts of pathway modulation. IL-5Rα protein levels were not assessed by Western blot in this preliminary study due to limited antibody availability and sample constraints, representing a limitation that will be addressed in future work. Moreover, the weak but statistically significant correlations observed in silico suggest a potential biological relationship, but they are not sufficient to establish causality.

To better contextualize these findings, it is important to consider how pathway-specific responses in HL-60 cells can provide insight into potential mechanisms of therapeutic intervention in immature promyelocytic-like AML.

Consistent with previous evidence indicating a pro-survival role of STAT3 signaling in AML cells, our findings show that Benralizumab significantly reduces STAT3 activation in HL-60 cells, while STAT5 protein levels remain unchanged. This selective modulation suggests that IL-5Rα targeting may preferentially interfere with STAT3-dependent survival pathways in immature promyelocytic-like AML models. It does not broadly suppress STAT signaling. Our findings align with data shown by Blechacz and colleagues [60]. In their research, the authors illustrated that the multikinase inhibitor Sorafenib significantly suppressed cell proliferation and induced apoptosis in the human AML cell line HL-60 by downregulating the constitutive activation of STAT3 [60]. Nevertheless, various studies have reported similar data, examining pharmacological and natural compounds that target STAT3. Examples include Fluacrypyrim, quercetin, a bioactive metabolite isolated from an endophytic fungus, and Jolkinolide B from the roots of Euphorbia fischeriana Steud [61,62,63].

Interestingly, the selective effect on STAT3 without affecting STAT5 may indicate that IL-5Rα blockade triggers targeted pro-apoptotic signaling rather than global disruption of JAK–STAT pathways.

Prolonged exposure of HL-60 cells to high-dose Benralizumab (50 μM for 3 days) resulted in increased pERK expression, suggesting the possible activation of compensatory MAPK signaling pathways in response to IL-5Rα blockade. This adaptive response may reflect a cellular attempt to counterbalance the pro-apoptotic effects induced by Benralizumab treatment. When cells were exposed to diphenyleneiodonium (DPI), a NADPH oxidase inhibitor, both ROS production and unfolded protein response (UPR) were inhibited [40].

In our experimental setting, increased expression of the NF-κB p65 subunit was observed only in HL-60 cells treated with the highest concentration of Benralizumab (50 μM), suggesting that strong IL-5Rα inhibition may trigger NF-κB-related stress or inflammatory signaling pathways. This effect was not detectable at lower drug concentrations, indicating a dose-dependent activation threshold.

At the highest concentration tested, we cannot exclude that receptor clustering phenomena or Fc-dependent interactions may have contributed to the observed signaling modulation. In the absence of Fc-silenced or isotype control antibodies, part of the molecular effects observed at 50 µM could reflect enhanced receptor aggregation rather than exclusively IL-5Rα-specific blockade. Future studies incorporating Fc-modified constructs or F(ab′)_2_ fragments will be useful to further dissect receptor-specific versus Fc-dependent contributions.

Bruton’s tyrosine kinase (BTK) is expressed in a subset of AML cases and can influence pro-survival pathways such as STAT3, NF-κB, and phosphoinositide 3-kinase/protein kinase B (PI3K/AKT). While BTK was not investigated in the present study, it represents a potential complementary mediator of leukemic cell survival alongside IL-5Rα signaling [64,65,66].

This dose-dependent NF-κB activation may reflect a threshold effect whereby mild IL-5Rα inhibition is insufficient to elicit stress signaling, whereas higher inhibition engages compensatory or pro-survival pathways.

To contextualize these NF-κB responses, previous studies have shown that pharmacological activators can also stimulate NF-κB–dependent differentiation in HL-60 cells.

However, different compounds are found to promote HL-60 differentiation by stimulating the NF-kB complex. For example, Brusatol, a quassinoid isolated from the fruit of *Brucea javanica*, induced the activation of the Rel/NF-κB pathway in HL-60 cells within 8 h [67]. This molecular phenomenon was attributed to the decrease in the total levels of the inhibitor of kappa B alpha (IkBα) and generation of the phosphorylated form of IkBα. Brusatol activity in HL-60 cells was blocked by SN50, a NF-kB translocation inhibitor [68]. Similarly, Daunorubicin increased the expression of NF-kB-related genes directly or by activating the tumor necrosis factor, which in turn induced the expression of NF-kB target genes [67]. A strong biological link between NF-kB and phosphoinositide 3-kinase/protein kinase B (PI3K/AKT) pathways has been found to contribute to the modulation of anti-apoptotic and multi-drug-resistant effects in AML cells. NF-kB and PI3/AK inhibitor peptides alone or synergistically led to a significant reduction in cell viability and stimulated apoptosis in a dose-dependent manner, representing a potential therapeutic for the treatment of AML [69,70]. In addition, in a variety of cellular conditions including HL-60 cells differentiating into macrophages by PMA, NF-κB activation was associated with p21 induction [71]. Moreover, p21 has been observed to induce cell cycle arrest in response to diverse stimuli, inhibiting cell cycle progression as a result [72]. The presence of damaging agents is a significant trigger for p21 activation, leading to this effect [73,74]. Also, p27 protein is involved in setting the direction of cell cycle. In this context, Yong-Ming Zhou and colleagues reported that the expression of p21 and p27 proteins increased in HL-60 cells treated with the proteasome inhibitor MG132 [75].

Our data indicate that p21 activation occurred exclusively in HL-60 cells exposed to high-dose Benralizumab for 3 days. In contrast, p27 levels progressively increased at both low and high concentrations, suggesting a dose-dependent regulation of cell cycle checkpoints. This differential regulation indicates that Benralizumab may induce distinct cell cycle checkpoint responses depending on drug dosage and exposure time. These responses may contribute to growth arrest and apoptotic commitment in AML cells. While mRNA validation could further strengthen these findings, the current study emphasizes protein-level modulation as a functional readout of pathway activation, consistent with its exploratory purpose.

Our results demonstrate that Benralizumab was associated with activation of apoptosis-related pathways in HL-60 cells, as indicated by Caspase-8/CASP8 p18 subunit (cleaved active form) induction. This finding is particularly relevant in the context of therapeutic strategies aimed at sensitizing AML cells to death receptor-mediated extrinsic apoptosis and supports the potential of IL-5Rα targeting as a pro-apoptotic intervention in immature myeloid leukemias. However, levels of the Bcl-2 protein were enhanced in a concentration dependent manner, indicating the capability of HL-60 cells to develop resistance to apoptosis and chemotherapy. This observation underscores the importance of considering combination strategies to overcome anti-apoptotic defenses in AML treatment. In this light, the development of novel pharmacological modulators of the anti-apoptotic Bcl-2 remains the preferred strategy to bypass chemoresistance and efficiently treat AML. Future studies will include parallel mRNA validation of apoptotic markers to further support the protein-level observations. From a translational perspective, IL-5Rα inhibition could be envisioned as a complementary strategy to established differentiation therapies such as ATRA or ATO [76]. By modulating survival and cell cycle-related signaling pathways, Benralizumab may potentially sensitize leukemic cells to differentiation-based treatments, a hypothesis that warrants future experimental investigation. Taken together, our results provide preliminary evidence that Benralizumab can influence survival- and cell cycle-related pathways in AML cells with promyelocytic features. These findings highlight IL-5Rα as a potential modulator of survival- and cell cycle-related pathways in AML cells with promyelocytic features, which warrants validation in models carrying the PML::RARA fusion.

## 5. Study Limitations

A limitation of this study is that experiments were performed in a single AML cell line, reflecting its exploratory nature. The quantities of Benralizumab available for these preliminary experiments were limited, which limited the scope of certain functional assays. Similarly, cell cycle analysis at the highest concentration (50 µM) was not included due to practical considerations associated with limited compound availability. These constraints, however, do not detract from the mechanistic insights obtained and provide a foundation for future studies.

While in silico analyses support the biological relevance of IL-5Rα in AML with promyelocytic features, a direct functional link between patient-derived transcriptomic data and in vitro responses in HL-60 cells was not established. Consequently, the findings should be considered preliminary and will require validation in primary AML/APL models. Nevertheless, in silico analyses suggest the potential relevance of IL-5Rα in AML with promyelocytic features, indicating that IL-5Rα–associated signaling pathways may be involved in early myeloid leukemias.

Furthermore, as the behavior of the bone marrow niche in vivo may differ from the in vitro model, the underlying mechanisms observed here may not fully capture the complexity of patient physiology. Additional major limitations include the absence of primary AML/APL patient samples, the lack of quantitative apoptosis assays, absence of metabolic viability assays, and the use of supra-physiological antibody concentrations. These aspects limit translational extrapolation and will require validation in primary leukemic blasts and in vivo models.

## 6. Conclusions

In conclusion, the present study shows that Benralizumab modulates selected signaling and cell cycle-related proteins in HL-60 cells, an immature myeloid leukemia model with promyelocytic-like features. Specifically, treatment was associated with reduced STAT3 activation, modulation of ERK phosphorylation, regulation of p21 and p27 expression, and cleavage of the caspase-8/CASP8 p18 subunit, suggesting activation of extrinsic apoptotic signaling pathways. These effects were observed under in vitro conditions and should be interpreted within the exploratory scope of this work.

Complementary in silico analyses of publicly available AML transcriptomic datasets indicate that IL-5Rα expression is detectable across specific AML subgroups. However, the observed correlations were generally weak despite statistical significance and do not establish causality. Importantly, the in vitro and in silico datasets were generated independently and were not integrated at the patient level.

**Figure 8:**
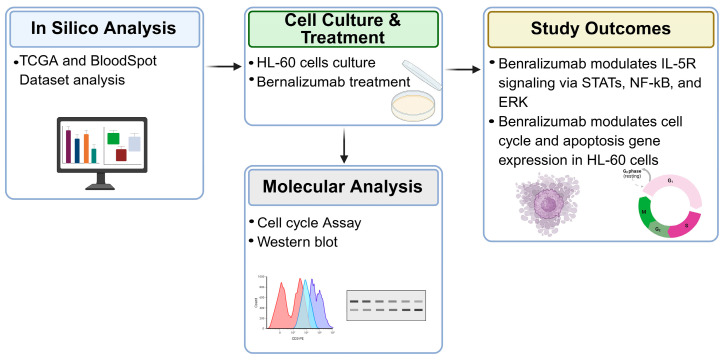
Benralizumab effects in AML: integrated approach and key outcomes. This flowchart provides an overview of the integrated experimental approach and its key findings, combining in silico data mining from TCGA and BloodSpot datasets with in vitro molecular validation in HL-60 cells. Western blot and cell cycle analyses confirmed that Benralizumab modulates critical intracellular signaling pathways including STAT3, pERK, and NF-κB. Collectively, these results demonstrate that Benralizumab treatment induces cleavage of Caspase-8/CASP8 p18 subunit, consistent with activation of extrinsic apoptotic signaling, and triggers cell cycle arrest, providing a biological rationale for its potential therapeutic efficacy in AML. Created in BioRender. Pelaia, C. (2026) https://BioRender.com/u430ro9 (accessed on 23 January 2026).

Overall, these findings provide preliminary mechanistic observations supporting further investigation of IL-5Rα-related signaling in immature myeloid leukemias. Additional studies in primary AML samples and comprehensive functional assays will be required to determine whether IL-5Rα targeting has biological or potential translational relevance in this context. A graphical summary of the study design and principal observations is provided in Figure 8.

## Data Availability

Additional data generated during the current study, including Western blot images and cell line experiments, are available from the corresponding author upon reasonable request.

## Supplementary Materials

The following supporting information can be downloaded at https://www.mdpi.com/article/10.3390/biomedicines14030652/s1, Figure S1: IL5-RA mRNA expression across AML subtypes. IL5RA mRNA expression levels measured as log_2_(value + 1) RNA-Seq CPM (counts per million) are shown across 451 clinical samples from the OHSU cohort [51]. Samples are categorized according to FAB classification and cytogenetic subtypes including acute megakaryoblastic leukemia, AML without maturation, AML not otherwise specified, acute monoblastic/monocytic leukemia, AML with maturation, AML with t(6;9)(p23;q34.1) DEK-NUP214, AML with minimal differentiation, AML with inv(16)(p13.1q22) or t(16;16)(p13.1;q22) CBFB-MYH11, acute promyelocytic leukemia (PML-RARA), and AML with t(8;21)(q22;q22.1) RUNX1-RUNX1T1. Each dot represents an individual patient while horizontal lines indicate the mean expression within each subgroup.; Table S1: Summary of hematopoietic cell populations and AML cytogenetic subtypes. This table provides a comprehensive overview of the cell populations and AML subtypes analyzed throughout the study, detailing their respective immunophenotypes and data sources. Data concerning human normal hematopoiesis ranging from hematopoietic stem cells (HSCs) and multipotential progenitors (MPPs) to lineage-specific progenitors and mature myeloid cells, were obtained from the GSE42519 dataset. These populations are defined by specific surface marker combinations as indicated in the immunophenotype column. Furthermore, human AML patient data were compiled from several independent cohorts, specifically GSE13159, GSE15434, GSE61804, GSE14468, and TCGA. The table summarizes major cytogenetic alterations including t(15;17), t(8;21), inv(16)/t(16;16), and various trisomies or deletions with statistical significance levels indicated where appropriate (e.g., *** *p* < 0.001; n.s., not significant).

## Abbreviations

ADCC	antibody-dependent cellular cytotoxicity
AKT	protein kinase B
AML	acute myeloid leukemia
APL	acute promyelocytic leukemia
ATO	arsenic trioxide
ATRA	all-trans retinoic acid
BM	bone marrow
CMP	common myeloid progenitor
DIC	differentiation induction chemotherapy
DMSO	dimethyl sulfoxide
ERK	extracellular signal-regulated kinase
FAB	French-American-British classification
FBS	fetal bovine serum
GMP	granulocyte-monocyte progenitor
HSC	hematopoietic stem cell
IL-5	interleukin-5
IL-5Rα	interleukin-5 receptor alpha
JAK	Janus kinase
MAPK	mitogen-activated protein kinase
MEP	megakaryocyte-erythroid progenitor
MPP	multipotent progenitor
NF-κB	nuclear factor kappa B
PB	peripheral blood
PI	propidium iodide
PI3K	Phosphatidylinositol 3-kinase
PM	promyelocyte
PMN	polymorphonuclear neutrophil
ROC	receiver operating characteristic
STAT	signal transducer and activator of transcription
TCGA	The Cancer Genome Atlas
UPR	unfolded protein response
