# Supplementary figures and images for: Preliminary Effects of Benralizumab in an AML Cell Model with Promyelocytic Features Expressing IL-5R: An Exploratory Proof-of-Concept Study
Source: Biomedicines. 2026 Mar 13;14(3):652. doi: 10.3390/biomedicines14030652 (PMC13023966; doi:10.3390/biomedicines14030652)

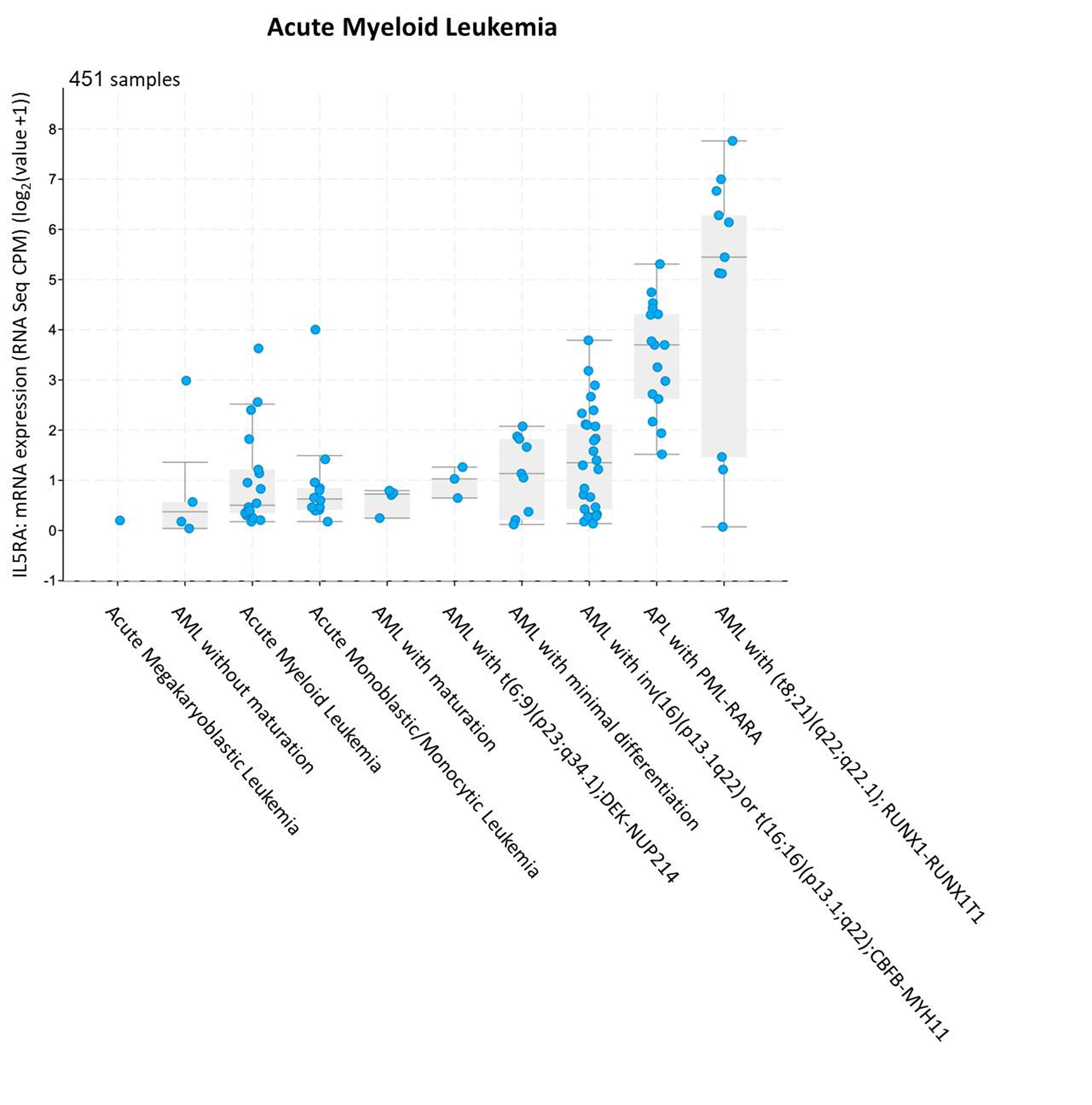

Supplement: Supplementary file 1 [file biomedicines-14-00652-s001.zip › Figure Suppl.S1.tif]
